# SARS-CoV-2, Influenza and Other Respiratory Viruses: Epidemiology, Burden of Disease, and Preventive Measures

**DOI:** 10.3390/vaccines13121234

**Published:** 2025-12-10

**Authors:** Emma Papini, Saverio Caini, Marco Del Riccio

**Affiliations:** Department of Health Sciences, University of Florence, Viale G.B. Morgagni 48, 50134 Florence, Italy

## 1. Introduction

Four years after the acute COVID-19 emergency, respiratory viruses circulate in overlapping seasonal patterns that repeatedly test surveillance systems and healthcare capacity. Policymakers now face several threats at the same time, including evolving SARS-CoV-2 variants, RSV epidemics, ongoing influenza antigenic drift and a rapidly changing prevention landscape that includes long-acting monoclonal antibodies for RSV, COVID-19 booster strategies across platforms, and long-standing adult and paediatric influenza vaccination programmes. Recent global analyses confirm that RSV remains a leading cause of acute lower respiratory infection and mortality in infants and young children, especially in low and middle income countries, and emphasise that newly available tools such as maternal RSV vaccination and long-acting monoclonal antibodies will only deliver their full impact if they can be implemented at scale and with equity [1,2,3]. According to recent modelling work, approximately 245,000 RSV-associated hospitalizations occurred annually in children under the age of five in Europe prior to the implementation of RSV immunisation programmes. Three quarters of these hospitalizations occurred in infants under the age of one year, with the highest rates occurring in those under two months [4]. Evidence along that continuum, such as outpatient RSV surveillance, pneumococcal disease dynamics under non-pharmaceutical interventions (NPIs), real-world RSV immunoprophylaxis, maternal influenza vaccination implementation, One Health spillover at the human–animal interface, and adolescent COVID-19 booster immunisation, is compiled in this Special Issue of *Vaccines*.

## 2. Overview of Published Articles

The first set of contributions discuss how we measure and interpret the burden of respiratory infections beyond using traditional hospital-based indicators. In contribution n.1, Boccalini and colleagues provide the first systematic overview of RSV in Italian paediatric outpatients, showing that among children 0–5 years presenting with an acute respiratory infection, RSV positivity ranges from about 18% to 41%, with estimates increasing from approximately 24% in 2001–2002 to around 41% in 2019–2020. This outpatient perspective fills a gap in burden estimates and offers critical denominators for evaluating preventive tools that mainly act outside the hospital. In contribution 2, Izquierdo et al., analyse invasive pneumococcal disease (IPD) in children in Catalonia, documenting that incidence fell from 11.0 to 4.6 cases per 100,000 person-years between the years 2018–2019 and 2021, with a 58% reduction. The authors demonstrate that by analysing incidence alongside emergency department visits and diagnostic requests, changes are revealed to be due to both decreased healthcare usage during the pandemic and actual changes in disease transmission resulting from NPIs. Extending surveillance beyond human clinical settings, Molini and colleagues found five dogs that tested positive for SARS-CoV-2 (one genome was classified as being of an Omicron BA.2 sub-lineage) among 500 sampled in Namibia (1%; 95% CI 0.33–2.32) (contribution 3). Although human-to-dog transmission appears to be the dominant route, the detection of infections is observed mostly in free-ranging outdoor dogs in a sub-Saharan setting, which underlines the feasibility and value of One Health surveillance in areas where empirical data have so far been scarce.

A second set of studies focuses on preventive tools and their performance when embedded in real-life programmes. In contribution 4, Consolati et al. describe a universal nirsevimab programme for newborns and infants in the Valle d’Aosta region during the 2023–2024 RSV season. Among 556 eligible infants, the overall coverage with a single dose reached 69%, rising to 86% in the most recent birth cohort. The proportion of infants hospitalised for RSV fell from 7.0% in 2022–2023 to 3.2% in 2023–2024, and no hospitalisations occurred among nirsevimab recipients, compared with 8.3% hospitalisations among eligible but non-immunised infants. At the maternal end of the life course, two studies examine influenza vaccination in pregnancy from complementary angles (contribution 5 and contribution 6). The survey by Lopatynsky-Reyes et al. examines 206 Mexican obstetricians and family physicians; it shows that, although almost all individuals are aware that influenza vaccination is recommended during pregnancy, more than a quarter do not recognise that influenza is more severe during pregnancy, over half do not know the potential complications of it, and more than half are unsure about the optimal timing of vaccination. Hence, providers with misperceptions about foetus safety or disease severity are less likely to report good vaccination practices (contribution 5). The study by McKeirnan and colleagues, based in a medically underserved county in Washington State with a high proportion of Hispanic or Latina residents, reports that only about one-quarter of respondents were vaccinated against influenza during pregnancy, identifying clear provider recommendation, being up to date on other vaccines and easier access to care as major facilitators of uptake, while non-vaccinated respondents frequently cited low perceived need, safety concerns and logistical barriers (contribution 6). Together, these three studies show that the potential impact of RSV and influenza vaccines depends not only on biological efficacy, but on coverage, equity and the behaviour of both providers and pregnant people in different health systems.

Finally, contribution 7, the study on Indonesian adolescents by Fadlyana et al., contributes to the evidence for the effective methods of sustaining protection against SARS-CoV-2 in younger age groups in countries that have relied heavily on inactivated vaccines. In adolescents between the ages of 12 and 17 years who had completed a two-dose CoronaVac series at least six months earlier, a single dose of the recombinant protein subunit vaccine IndoVac increased neutralising antibody geometric mean titres from approximately 303 to 2661 IU/mL at 14 days post-booster, an almost nine-fold rise, with titres remaining above the baseline at 3, 6 and 12 months and a reactogenicity profile dominated by mild local pain and myalgia. No vaccine-related serious adverse events were reported. These data, importantly, help to bridge the immunogenicity and safety gap in heterologous boosting in adolescents and illustrate how domestically produced protein-based vaccines can offer scalable options in middle-income settings in which cold-chain constraints and supply limitations may hinder the broader use of mRNA platforms.

## 3. Discussion

The contributions in this Special Issue span across a range of pathogens, populations and settings, but they converge on a common theme: the need to align powerful preventive technologies with the realities of health systems, healthcare provider practice and community experience. As RSV tools emerge, COVID-19 booster strategies evolve and maternal influenza programmes aim to close persistent gaps, the types of evidence presented here will be essential for guiding prioritisation, targeting and delivery so that the benefits of vaccination reach those who most require them. At the same time, the studies in this Special Issue underscore that, despite the clear progress achieve, the field of respiratory vaccination still suffers from structural gaps that are important to bridge. For example, outpatient RSV surveillance in Italy remains fragmented: the systematic review included demonstrates that only six eligible studies over two decades of research have used heterogeneous clinical definitions and testing strategies, often without including stable denominators for unique patients or visits, therefore limiting the comparability of RSV positivity across time and place and emphasizing the value of standardising primary care protocols such as those proposed by van Summeren and colleagues [5]. Then, the Catalan IPD analysis illustrates how much insight can be gained when microbiological data are explicitly paired with healthcare seeking and testing volumes, allowing for changes in incidence by serotype, age and quarter to be interpreted as real epidemiologic shifts. Furthermore, with regards to the human–animal interface, the Namibian dog survey demonstrates the feasibility of molecular and genomic surveillance in low-resource peri-urban settings, but also how sparse and cross-sectional One Health data still are, with no paired human sampling. 

Beyond this Special Issue, phase 3 maternal RSV vaccine trials, rapid policy uptake by international agencies and early real-world evaluations of nirsevimab in several countries are already showing that these infant protection strategies have potential to substantially reduce RSV hospitalisations, while also revealing persistent challenges in coverage and equity [6,7,8,9].
